# A Systematic Review of Diet Quality Index and Obesity among Chinese Adults

**DOI:** 10.3390/nu13103555

**Published:** 2021-10-11

**Authors:** Isma’il Kadam, Sudeep Neupane, Jingkai Wei, Lee Ann Fullington, Tricia Li, Ruopeng An, Li Zhao, Amy Ellithorpe, Xinyin Jiang, Liang Wang

**Affiliations:** 1 Program in Biochemistry, Graduate Center of the City University of New York, New York, NY 10016, USA; ikadam@gradcenter.cuny.edu; 2 Department of Health and Nutrition Sciences, Brooklyn College of the City University of New York, Brooklyn, NY 11210, USA; ae1741@nyu.edu; 3 Department of Public Health, Robbins College of Human Health and Sciences, Baylor University, Waco, TX 76711, USA; sudeep_neupane1@baylor.edu (S.N.); Tricia_Li@baylor.edu (T.L.); 4 Department of Epidemiology and Biostatistics, Arnold School of Public Health, University of South Carolina, Columbia, SC 29208, USA; jwei@mailbox.sc.edu; 5 Library Department, Brooklyn College of the City University of New York, Brooklyn, NY 11210, USA; LAFullington@brooklyn.cuny.edu; 6 Brown School, Washington University, St. Louis, MO 63130, USA; ruopeng@wustl.edu; 7 West China Fourth Hospital, West China School of Public Health, Sichuan University, Chengdu 610041, China; zhaoli@scu.edu.cn

**Keywords:** diet quality, obesity, body weight, Chinese

## Abstract

Diet quality scores are designed mainly based on Western-style dietary patterns. They were demonstrated to be good indicators of obesity in developed but not developing countries. Several diet quality scores were developed based on the Chinese dietary guidelines, yet no systematic review exists regarding how they were related to obesity. We searched research articles published between 2000 and 2021 in PubMed, CINAHL, and Scopus databases. Both cross-sectional and prospective studies that examined the relationship between a diet quality score and weight, body mass index, obesity, or waist circumference conducted in a Chinese population were selected. From the 602 articles searched, 20 articles were selected (12 are cross-sectional studies and 8 are prospective cohort studies). The relationship between internationally used scores and obesity was inconsistent among studies. Scores tailored to the Chinese diet demonstrated a strong relationship with both being underweight and obesity. The heterogeneity of the populations and the major nutrition transition in China may partially explain the discrepancies among studies. In conclusion, diet quality scores tailored to the Chinese diet may be associated with both undernutrition and overnutrition, as well as being underweight and obesity outcomes.

## 1. Introduction

Obesity, or excess adiposity, is a leading public health problem worldwide that results in significant medical burdens [1]. A U.S. based study estimates that individuals who are obese spend 76% more on healthcare compared to those of normal weight [2]. Studies have documented a link between obesity and various chronic diseases such as type 2 diabetes [3], cardiovascular diseases [4], cancer [5], and chronic kidney disease [6]. 

Obesity is a complex disease that results from the interactions of various risk factors that influence the balance of energy intake and output, such as genetics [7], physical activity [8], gut microbial composition [9], dietary intake, and the obesogenic food environment, where the modern food system makes nutrient-poor, ultra-processed food more accessible and affordable [10,11]. The socioeconomic and sociocultural characteristics of people affect their vulnerability to the obesogenic risk factors [12]. Different policies have been established or proposed to curb the obesity epidemic, such as taxation on unhealthy food purchases [13], food pricing strategies [14], and nutrition education within food assistance programs [15]. 

One of the cornerstones to address the obesity epidemic is through dietary intervention. Food groups such as whole-grain, fruit, nut, legume, and fish were found to be associated with a reduced risk of obesity, whereas refined grains, red meat, and sugar-sweetened beverages were associated with increased risk [16]. Diet quality scores are developed to guide food consumption behaviors and assess the quality of the overall diet in the population. A systematic review by Ashgari et al. examined studies that assessed the relationship between diet quality scores and body mass index (BMI) and other indicators of obesity [17]. They found that diet quality assessed using different scores were, in general, inversely associated with BMI in developed countries, yet results were much more variable in developing countries. 

The complex relationship between diet quality scores and BMI in developing countries is likely related to the dual burden of both undernutrition and overnutrition in these countries. Likewise, China has been under a drastic transition in dietary habits during economic growth and development in recent decades, leading to rapid increases in the consumption of edible oils, animal source foods, sugar-sweetened beverages, and food away from home [18]. Accordingly, the prevalence of overweight and obesity rose rapidly in the country in the past several decades [19]. Meanwhile, undernutrition is still a concern for vulnerable groups, such as older adults in China [20]. Diet quality scores may provide a convenient tool to screen for those at risk of under- or over- nutrition. However, diet quality scores used internationally were designed mainly based on Western-style dietary patterns, which are substantially different from traditional dietary patterns in Chinese populations [21,22]. Therefore, it is critical to develop and evaluate the validity of culturally sensitive diet quality scores and test their ability to predict weight and adiposity in Chinese populations. 

Several diet quality scores have been designed specifically for the Chinese population based on the Dietary Guidelines for Chinese Residents (DGC) and the Chinese Food Pagoda (CHFP), such as the Chinese Healthy Eating Index (CHEI) [23] and Dietary Balance Index (DBI) [24]. Other scores used for international populations (e.g., Alternative Mediterranean Diet Score [aMED], Dietary Approaches to Stop Hypertension [DASH], and Alternative Healthy Eating Index [AHEI]) may also be compatible with Chinese populations [5]. This systematic review aimed to assess their contents, similarities, and differences, and their associations with BMI and other indices of obesity in Chinese adults. 

## 2. Materials and Methods

### 2.1. Study Design

Cross-sectional studies, longitudinal studies, nested case-control studies, and randomized controlled trials (RCTs) were eligible to be included while reviews, meta-analyses, and editorials were excluded.

### 2.2. Participants

Studies were included if they enrolled non-pregnant, non-lactating Chinese adults aged over 18 or included a subgroup of Chinese adults that were analyzed separately. Studies were excluded if they only included children, pregnant or lactating women, non-Chinese populations, or multiethnic populations that did not include separate analyses on the subgroup of Chinese adults.

### 2.3. Exposure

The exposure of interest was any standard dietary index that assessed the overall quality of the diet, such as the AHEI, Diet Quality Index-International (DQI-I), Diet Quality Divergence Index (DQD), CHEI, DBI, aMED, and DASH. Studies investigating single nutrients, food groups, or dietary patterns that were not based on a priori dietary index were excluded.

### 2.4. Outcomes

The primary outcome of interest was overweight and obesity as assessed by BMI. The BMI cut-offs were <18.5 kg/m^2^ for underweight, 18.5–23.9 kg/m^2^ for normal weight, 24–27.9 kg/m^2^ for overweight, and ≥28 kg/m^2^ for obesity for Chinese cohorts, unless specified otherwise in individual studies. These cut-offs were established by the Working Group on Obesity in China on the basis that obesity-related metabolic risk occurs at a lower BMI in Asians [25,26]. Secondary outcomes of interest were measurements of central obesity such as waist circumference (WC), waist-to-hip ratio, as well as body weight and weight change over time. Studies were included if they contain clear statistical analyses of the relationship between the exposures and outcomes of interest with the odds ratio (OR) and/or *p*-value presented.

### 2.5. Search Strategies

We selected PubMed, Cumulative Index of Nursing and Allied Health (CINAHL), and Scopus as our databases to conduct comprehensive searches for research articles published between 1 January 2000 and 30 June 2021. The keywords used in the search strategies included “China” and “Chinese” for the population, and, for the exposures, the keywords including “diet quality”, “nutrition survey”, and “dietary index” were used. We did not include searches for outcomes in order not to over-restrict our results [27] in PubMed and CINAHL. However, for Scopus, we did include keywords for outcomes, including “obese”, “waist circumference”, and “overweight.” For PubMed, searches used a combination of MeSH and keywords. In addition, citation tracing searches were performed on studies that met inclusion criteria to further ensure the research comprehensiveness and locate studies that may not have been retrieved using the predefined search algorithms. Search results were imported to Endnote (Clarivate Analytics, London, UK) for study selection.

### 2.6. Study Selection

We first removed duplicates identified across databases using Endnote. Thereafter, two authors screened the manuscripts identified through the electronic database search based on their titles and excluded those found to be irrelevant. These two authors then conducted an abstract and full-text review of the remaining papers based on the inclusion and exclusion criteria. Disagreements between the two authors were resolved by consensus or a third person’s decision if consensus was not reached.

### 2.7. Study Quality Assessment

We assessed the quality of studies using the National Institutes of Health Quality Assessment Tool for Observational Cohort and Cross-Sectional Studies [28]. The assessment tool includes 14 equally weighted criteria and thus the highest possible score indicating high-quality study is 14. Scores less than 7 indicated a high risk of bias, 7–10 indicated a moderate risk, and 11–14 was considered as low risk. 

## 3. Results

**Figure 1:**
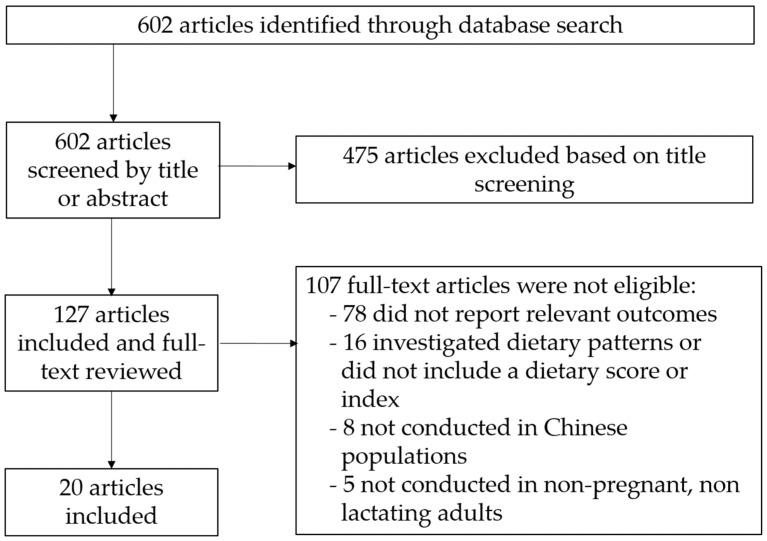
Flowchart selection.

We identified 602 articles from the initial database search (Figure 1). After screening based on title or abstract, 127 papers remained and were included in the full-text review. Of those, 107 were found to have irrelevant outcomes, topics, or study populations and were therefore excluded from the review. After the search and selection process, 20 articles were found to meet all inclusion criteria and were included in the review (Table 1). Of these 20 articles, 12 were cross-sectional studies, and 8 were prospective cohort studies. There were no RCTs that met the inclusion criteria. Fourteen of the articles used a diet quality index designed or modified for the Chinese population such as the tailored AHEI (tAHEI), the CHEI, the adherence to CHFP, the China DQI, DBI, and the dietary diversity score (DDS); five of them included diet quality scores used internationally such as the HEI and AHEI, DASH and aMED scores; and one study included both tailored and internationally used scores. All but one of the five studies that only included internationally used scores were conducted in Chinese populations outside mainland China, while all but one of the 14 studies that used tailored scores were conducted within mainland China. All studies included BMI as an outcome, five of them also assessed the risk of being underweight or overweight and obese, and five of them included WC as an outcome. Four studies had less than 1000 participants, 11 studies had 1000–10,000, and 5 studies had over 10,000 participants. Twelve studies used 24-h dietary recall, seven used a food frequency questionnaire (FFQ), and one used both dietary recall and FFQ to collect dietary information. 

### 3.1. HEI and Obesity-Related Outcomes

Among the 20 included articles, nine included a version of the HEI as an assessment of diet quality. The original HEI measures diet quality according to the Dietary Guidelines for Americans (DGA) and has updated versions with new DGAs [29]. The AHEI incorporates some HEI components and additional foods and nutrients relevant to the risk of chronic disease (Table 2) [29]. Six out of the nine studies assessed how AHEI or modified AHEI were associated with BMI. Two cross-sectional studies identified an inverse relationship between AHEI and obesity. Cheung et al. demonstrated that AHEI-2010 had an inverse association with obesity (OR: 0.95, 95% CI: 0.91–0.99, *p* = 0.02) in 211 Hong Kong Chinese adults with type 2 diabetes [30]. Whitton et al. also found that AHEI-2010 was inversely associated with BMI in 4617 Chinese participants within a multiethnic cohort in Singapore [31]. Three other studies found no association between AHEI or a modified or tailored version of AHEI (mAHEI) and BMI. Neelakantan et al. conducted a nested case-control study among 751 participants who experienced acute myocardial infarction and 1443 matched controls. BMI did not differ among AHEI quartiles in control participants (*p* = 0.43) [32]. Chou et al. assessed 436 community-dwelling elders in Taipei, China, and did not find significant differences in BMI between mAHEI tertiles either (*p* = 0.26) [33]. Wang et al. developed a tailored AHEI (tAHEI) to assess diet quality in the China Health and Nutrition Survey (CHNS) longitudinal study. They analyzed data from 4734 participants who had over two waves of dietary data from 1991 to 2006 in the study. Their results suggested that there were no differences in baseline BMI in low versus high tAHEI score tertiles [34]. However, when they analyzed all participants who participated in the 2006 CHNS, the top quintile of tAHEI was associated with higher BMI and WC (both *p* < 0.001) than the bottom quintile in men [35]. In this study, they also developed a Chinese Dietary Guidelines Index (CDGI) based on the dietary recommendations in the DGC. Men in the top quintile of CDGI demonstrated 35% lower odds of abdominal obesity (OR 0.65; 95% CI 0.44–0.97) than those in the bottom quintile. However, there were no significant differences in BMI across the quintiles of CDGI.

One study by Gao et al. examined the association of obesity outcomes with the original HEI-90 and the updated 2005 HEI (HEI-05) in the Multi-Ethnic Study of Atherosclerosis (MESA), which contained a subgroup of Chinese American participants (*n* = 790) [36]. The study recorded BMI at baseline and an 18-month follow-up. It was demonstrated that the HEI-90 z score was not associated with BMI at baseline, yet each unit increment of this score was linked with a decrease of 0.37 kg/m^2^ of BMI in follow-up (*p* = 0.008). In contrast, HEI-05 was associated with lower BMI at both baseline (*p* = 0.038) and follow-up (*p* = 0.005). Each unit increment in the HEI-05 z score was linked with a BMI decrease of 0.26 kg/m^2^ and 0.43 kg/m^2^ at these two time points, respectively. Additionally, each one-unit increment of HEI-05 was associated with a 3% decreased obesity risk. However, there was no association between HEI and WC in Chinese participants, though an association was found in white participants. 

Two studies reported the relationship between the CHEI and BMI. The CHEI adopts the scoring methodology of HEI but bases its scoring on the DGC-2016 (Table 2) [23]. Yuan et al. developed this index and examined its relationship with BMI in a cross-sectional study using 2011 CHNS (*n* = 14,584) [23]. Their results suggested that, compared to people with normal weight, people who were underweight had lower CHEI scores [coefficient = −2.73, 95% CI: −3.27, −2.20, *p* < 0.001]. However, there were no significant differences in CHEI between normal weight and overweight or obese participants. Liu et al. analyzed the relationship between CHEI scoring change over time and obesity outcomes using data from 6398 CHNS participants who participated in at least four waves of the survey in 1997–2015 [37]. They found that those who had low CHEI at baseline and remained low over time were the least likely to have a normal BMI at baseline (*p* < 0.001).

Overall, the relationship between an HEI-based score and weight or obesity was inconsistent among the studies. The inconsistency may be attributed in part to the heterogeneity of the populations investigated in different studies. The three studies that showed an inverse relationship between an HEI-based score and BMI and obesity were conducted in Chinese populations from more developed countries or regions. Specifically, the Gao et al. study was conducted in the American Chinese population in the U.S. [36], the Cheung et al. study [30] was conducted in Hong Kong, China, whereas the Whitton et al. [31] study was conducted in Singapore. The inverse relationship between the HEI-based scores and BMI was consistent with the prominent public health concern in developed countries and regions that easy and affordable access to high-fat, high-sugar, ultra-processed food has promoted the development of an obesogenic dietary environment [38]. 

All studies using an HEI tailored to the Chinese population (tAHEI and CHEI) were conducted using participant data from different waves of CHNS. However, the results were inconsistent. Wang et al. included the most CHNS participants recruited at different periods (1991–2006) [34]. These periods captured the various stages of nutrition transition in China. The survey also captured regions of different degrees of development in China [34]. This heterogeneity within the cohort may partly explain the lack of association between tAHEI and BMI in this study. The positive relationship between tAHEI or CHEI and BMI in the Wang et al. [35] and Yuan et al. [23], especially the lower risk of underweight with higher CHEI, likely indicated that undernutrition was still a significant concern in the CHNS cohorts recruited during the nutrition transition in China. 

### 3.2. Adherence to the CHFP and Obesity-Related Outcomes

Two studies examined how adherence to the CHFP was related to BMI. The CHFP embodies the principles of the DGC and specifies the recommended proportion of different food groups in the diet [39]. The CHFP score was based on 10 dietary components and the scoring method of the HEI [40]. Nguyen et al. investigated how CHFP scores were related to colorectal cancer risk in two ongoing population-based prospective cohort studies, including the Shanghai Women’s Health Study (SWHS) and the Shanghai Men’s Health Study (SMHS) containing 72,445 women and 60,161 men, respectively [41]. It was demonstrated that, at baseline, BMI did not differ (*p* = 0.11) between quartiles of CHFP score among participants. Wang et al. investigated how CHFP scores were associated with cancer outcomes in 3450 breast cancer survivors from the Shanghai Breast Cancer Survival Study [42]. In this study, participants within the highest quartile of CHFP scores were more likely to have lower BMI (24.0 ± 3.3 versus 24.6 ± 3.8 kg/m^2^) compared to participants within the lowest CHFP score quartile.

The mixed results in these two studies preclude the possibility to evaluate the validity of this index as a marker or predictor of weight control in Chinese populations [41,42]. While this index was not associated with BMI in a study among healthy people [41], it was negatively associated with BMI in breast cancer survivors [42]. It remains for further investigation whether the health status of an individual plays a role in modifying the sensitivity of this index in association with weight status. 

### 3.3. The China Diet Quality Index (DQI) and Obesity-Related Outcomes

Two studies explored the relationship between the China DQI and obesity. Stookey et al. developed the China DQI tailored to measure diet quality in Chinese residents [43] (Table 2). The DQI components were selected based on world reference values, Chinese Recommended Daily Allowances (RDA), and CHFP. A DQI score converging to 0 is considered as a more balanced diet. Stookey et al. used the 1991 panel of CHNS, which included 7450 participants, to examine the validity of the index. They found that, with each unit increase in the DQI score, the risk of underweight decreased significantly (OR = 0.995, 99% CI: 0.991 ± 0.999, *p* = 0.02) and the risk of overweight increased significantly (OR = 1.008, 99% CI: 1.005 ± 1.013, *p* < 0.0001) [43]. In a later study, Huang et al. examined the DQI and obesity relationship using data from four waves (2004, 2006, 2009, and 2011) of CHNS (*n* = 13,833 for those included in the regression analysis) [44]. They demonstrated that the DQI score was positively associated with BMI in overweight [coefficient 1.513, 95% CI (0.892, 2.134), *p* < 0.001] and obese [coefficient 1.883, 95% CI (0.962, 2.805), *p* < 0.001] individuals compared to normal-weight individuals [44]. Overall, a higher China DQI score (i.e., a less balanced, lower quality diet) may be a good indicator that reflects the overnutrition of people who are overweight or obese in Chinese populations.

### 3.4. The Dietary Balance Index (DBI) and Obesity-Related Outcomes

Three studies assessed how DBI was related to obesity. The DBI was developed from the China DQI. It evaluates diet quality by four different indicators, including Total Score (TS), which converges to 0, Lower Bound Score (LBS), which assesses inadequate intake, Higher Bound Score (HBS), which assesses excessive intake, and Diet Quality Distance (DQD), which evaluates the degree of imbalance of the diet (Table 2) [45]. Xu et al. demonstrated in their study of 2745 older adults (aged > 60) in the CHNS 2009 cross-sectional cohort that DBI-07 differed by BMI [46]. Specifically, those who were underweight had higher LBS, lower HBS, and lower TS (*p* < 0.01). Zang et al. assessed the DBI in 1680 participants of the Shanghai Diet and Health Survey (SDHS) and found that obese people had higher DQD scores than people with normal weight [coefficient 1.16, 95% CI (0.00, 2.36), *p* = 0.049] [47]. Zhang et al. assessed the DBI in 738 participants aged 50–77 years in the 2010–2012 National Nutrition and Health Survey in Yunnan province, southwest China [48]. They found that being underweight was associated with a higher LBS score (*p* < 0.05) and that DQD scores were higher among people who were underweight (*p* < 0.05) [48].

Recently, Zhou et al. developed a standalone diet quality divergence Index (abbreviated as DQD(2) to distinguish from the DQD above) to assess the deviation of the diet from the CHFP [49]. They assessed data from 30,626 adults in the CHNS and demonstrated that, as BMI increased, the DQD(2) increased. The normal-weight group had lower DQD(2) scores than the obesity group (*p* < 0.05) and the overweight group (*p* < 0.01) [49]. In summary, both underweight and overweight/obesity were associated with suboptimal DQI in Chinese populations.

### 3.5. Food Diversity and Obesity-Related Outcomes

Two studies assessed how food diversity was associated with obesity-related outcomes. Tian et al. analyzed the food diversity of 17,825 participants from four waves (2004, 2006, 2009, and 2011) of the CHNS using a Dietary Diversity Score (DDS) (Table 2). They found a positive association between DDS and overweight only in men [OR = 1.09 (95% CI 1.03–1.17)] [50]. Zhao et al. developed a Chinese Healthy Food Diversity (HFD) index using the Chinese Urban Adults Diet and Health Study (CUADHS) as the primary dataset (*n* = 1520) and 2009 CHNS as the validation dataset (*n* = 2398) [51]. HFD scores were negatively associated with WC in the CHNS but not the CUADHS dataset, while this index was not significantly different between BMI categories in both datasets [51]. The limited number of studies and inconsistent results preclude a conclusion on the relationship between food diversity and obesity in Chinese populations. It should be noted that DDS was composed of the variety of both healthy and unhealthy foods, while HFD measured the variety of healthy food only. Therefore, it seems that, without the premise of healthy food, increasing dietary diversity may not necessarily be associated with better weight outcomes.

### 3.6. Dietary Approaches to Stop Hypertension (DASH) and Alternate Mediterranean Diets (aMED) Scores and Obesity-Related Outcomes

DASH and MED are recognized as healthy diets worldwide (Table 2). Three studies also assessed at least one of these diets’ relationship with obesity. Jia et al. reported in a cross-sectional study of 1320 adults in Inner Mongolia, China that DASH scores were associated with a lower risk of abdominal obesity (assessed with waist circumference, WC) with the OR in the highest versus lowest tertile being 0.71 (95% CI 0.53, 0.96, Ptrend  =  0.03) [52]. aMed adherence was associated with a lower risk of excessive WC (OR 0.63, 95% CI 0.47, 0.87; Ptrend  =  0.005) and lower risk of obesity (OR 0.60, 95% CI 0.44, 0.81; Ptrend  =  0.02) [52]. The Whitton et al. study mentioned above also examined the relationship between DASH or aMED and obesity [31]. Their results demonstrated a negative association of DASH with obesity (*p* < 0.05), yet aMED was not related to obesity outcomes [31]. The Cheung et al. study mentioned above did not find any association between DASH scores and obesity risk, although aHEI was demonstrated to be inversely associated with obesity in the same study [30]. This study did not find an association between the Diet Quality Index–International (DQI-I) and obesity [30]. Overall, the association of DASH and aMED with weight status was not consistent across studies of Chinese populations. 

### 3.7. Quality of Studies

Only two studies (10%) included in this review were considered to have a low risk of bias, 13 studies (65%) were determined as of moderate risk of bias, and 5 studies (25%) were considered as having a high risk of bias (Supplementary Table S1). The studies with an increased risk of bias were all cross-sectional studies that did not have appropriate confounding factors controlled for in assessing the dietary score and weight or obesity outcomes. Twelve out of the 13 studies with a moderate risk of bias were cross-sectional studies that could not discern the chronological order between the dietary scores and weight or obesity outcomes. Studies with a low risk of bias were both longitudinal studies. Overall, the cross-sectional design of most studies contributed to the higher risk of bias. The lack of proper adjustment for confounders may have contributed to the higher percentage (40%) of studies with a null result in the studies considered as having an increased risk of bias versus those with a moderate risk of bias (15%).

## 4. Discussion

This review systemically analyzed existing studies that explored the relationship between diet quality scores and obesity-related outcomes in Chinese populations. As adherence to a priori high-quality dietary pattern is increasingly recommended to improve overall healthfulness during the drastic nutrition transition in China, this review evaluated whether the quality of a diet, independent of calorie content, has implications in weight management and obesity reduction. Both internationally accepted, non-culture-specific diet quality scores or tailored scores to the Chinese diet were tested in several cohorts of Chinese participants, mainly with a cross-sectional design, yet the relationships between these scores and obesity-related outcomes varied. The inconsistencies may be attributed in part to the heterogeneity of the cohorts and the need for tailored diet quality assessment according to the characteristics of specific populations.

A significant source of heterogeneity lies in the geo-economic and cultural differences among cohorts, specifically, between Chinese adults within versus outside mainland China. Three out of four studies that used AHEI or HEI in Chinese populations outside mainland China demonstrated an inverse association between the dietary index and BMI [30,31,36], consistent with the concern of an obesogenic environment typical for developed countries and regions. 

Consistently, the systematic review by Asghari et al. has previously demonstrated that HEI reflected a reduced risk of obesity in studies conducted in developed but not in developing countries [17]. In cohorts within mainland China, the diet quality has a divergent relationship with weight status, with the diet quality scores being associated with both underweight and overweight/obesity in different studies [23,43,53]. This is typical for developing countries during drastic nutrition transitions. The dual burden of undernutrition and overnutrition was clearly reflected in studies using data from the CHNS, which included waves of cross-sectional cohorts ranging from 1990 to 2015 that coincided with the rapid economic development and nutrition transition of China. Both underweight and overweight/obesity were associated with worse dietary scores (e.g., China DQI, DBI-07, CHEI) in studies based on different waves of CHNS data. The dichotomized association of dietary scores with both under- and overnutrition observed in different waves of participants also suggests that the drastic nutrition transition in China may have altered the relationship between diet quality and obesity over time.

The nutrition transition in China is a result of rapid economic development after the reform and opening policy was implemented in the late 1970s. The rapid economic growth in China fuels the modernization of the food system and enhances the purchasing power of people in China. Accompanying with the increased affordability and accessibility of food, people in China experienced Westernization of dietary intake, characterized by significant increases in edible oil and meat consumption, snacking behavior and sugar-sweetened beverage consumption, and decreases in cereal grains and legumes that are emphasized in the traditional Chinese diet [56,57,58]. However, the nutrition transition is not identical for everyone, and several factors influence the level of adoption. Older adults have greater adherence to the traditional Chinese dietary pattern (e.g., cereal-rich and limited in meat) [46]. This may partly explain the higher risk of undernutrition of this group associated with lower diet quality scores. Rural versus urban areas have different levels of nutrition transition partly attributable to the different levels of economic development. The more economically developed urban areas had greater levels of nutrition transition as demonstrated by changes such as increased snacking behavior and fried food consumption [58,59]. Overnutrition and obesity become the main concerns in those areas. However, it should be noted that the gap between rural and urban areas has gradually narrowed. The Scientific Research Report on DGC 2021 demonstrates that the dietary structure of rural residents has been greatly altered with carbohydrate-derived caloric intakes decreasing from 70.1% in 1992 to 55.3% in 2015, and the protein provided by animal food increasing from 12.4% to 31.4% [60]. The Chinese Centers for Disease Control and Prevention (CDC) research data show that the obesity rate in rural areas was about half of that in urban areas (12.2% versus 6.3%) in 2002; by 2012, the rural obesity rate grew to 10.4%, which was very close to the 13.2% in urban areas [61]. At the individual level, those with a higher socioeconomic status (SES) have accelerated nutrition transition and obesity becomes the primary concern. In contrast, those with a lower SES have a slower change and a higher risk of undernutrition and being underweight [18,62]. These differences in nutrition transition among different groups suggest the need to choose an appropriate diet score according to its sensitivity to either under- or over-nutrition. 

A potential concern of using internationally used, non-culture-specific dietary scores such as HEI was that those scores were not developed based on the DGC. There were substantial differences between the Chinese and Western diets. These differences were also reflected in their dietary guidelines, such as a higher recommended amount of soy and a lower recommended amount of dairy in the CDG versus the DGA [63]. An HEI-based score showed inconsistent relationships with weight status in different Chinese cohorts, as mentioned above. Several studies also used DASH and aMED to assess diet quality. These scores were not based on the official dietary guidelines of specific countries, but rather on two well-accepted high-quality diets, the DASH and Mediterranean diets. Previous studies have shown that adopting these diets improved cardiometabolic outcomes and in non-Chinese populations [64,65,66,67,68]. However, the results were inconsistent among the few studies that assessed how these indices were associated with BMI in Chinese cohorts, suggesting that a tailored score to the Chinese diet may have better sensitivity to serve as an indicator of weight status in Chinese. 

Several indices tailored to the Chinese diet were developed according to the CDG and CHFP, such as the CHEI, CHFP score, China DBI, and DDS. DBI was developed with a set of scores (LBS, HBS, TS, and DQD) that make it convenient to assess both under and over nutrition [45]. Indeed, in some Chinese populations, such as among older adults, undernutrition was the main concern, such as what Xu et al. reported regarding the worse DBI in underweight elders [46]. Zhang et al. also showed in a cohort with late middle-aged and older adults in the Yunnan province of China that DBI was related to the risk of being underweight [48]. A study conducted on the second wave of the nationally-representative Chinese Health and Retirement Longitudinal Study (CHARLS) estimated that the prevalence of malnutrition among elderlies in China was 12.6%, or over 20 million elderlies [20]. Malnutrition in older adults has been associated with adverse functional and mortality outcomes [69]. Therefore, using a diet quality score to assess and predict malnutrition and weight loss in older adults may help identify those that need intervention and assistance. However, two other studies that enrolled Chinese adults of different ages showed that the more imbalanced the diet was, as assessed by the DQD score, the higher the BMI and risk of obesity [47,49]. Therefore, the DBI may be a good indicator of malnutrition (both under- and over- nutrition) in Chinese adults, yet its specific relationship with weight status may depend on characteristics of the cohorts (e.g., age, health status, socioeconomic status). 

Besides the DBI, the China DQI was another index that consistently demonstrated an association with overweight in two Chinese cohorts, suggesting the potential use of this index to assess overnutrition in Chinese populations [43,44]. 

Food diversity is differentially defined in different studies and may be a double-edged sword for weight control. Food diversity was demonstrated to be positively associated with BMI in studies of non-Chinese populations and summarized in the Asghari et al. review [17]. A potential explanation was that diversity could be attributed to both varieties of healthy and unhealthy foods. It is also possible that people tend to eat more when there is a greater variety of food, leading to a higher total energy intake [70]. The one study that we reviewed used a similar food diversity score to those used in other studies, which included the variety of both healthy and unhealthy food. Consistent with previous findings, it showed that food diversity was positively associated with overweight though only in men [50]. The other study developed a score that assessed the variety of healthy food specifically (HFD) and did not observe an association between HFD and weight while observing a negative association between the index and WC [51]. These studies corroborate the notion that not just the variety of foods eaten, but also what is being consumed matters for obesity reduction. 

Overall, diet quality scores tailored to the Chinese population are associated with either being underweight or overweight/obese in Chinese adults. With the dual burden of undernutrition and the rising obesity epidemic, the Chinese government has implemented various policies and programs to reduce both underweight and obesity. For example, the Ministry of Health released the Nutrition Improvement Work Management Approach to promote nutrition and health of Chinese residents [71]. The General Office of the State Council released the National Nutrition Plan (2017–2030) in 2017 [72] and the Department of Planning released the Health China Action in 2019 [73] which propose rational dietary behaviors and strengthening dietary and nutrition guidance for the whole population. These initiatives focus on encouraging the reduction of salt, oil, and sugar intakes in every sector, including the food industry, the cafeteria of enterprises and institutions, and families. They also require governmental departments to develop and implement relevant regulations and standards, and implement nutrition interventions for vulnerable populations in impoverished areas. Although this systematic review did not include studies conducted in children, it should be noted that the obesity rate of children and adolescents in China is rising alarmingly [74]. Several policies have been proposed to address this significant public health concern such as accelerating the development of standards to limit production and sale of high-sugar foods, revising the general rules for nutrition labeling to include mandatory labeling of sugar in prepackaged foods, and enhancing the nutrition education about added sugar intake restriction [75]. 

The establishment of the DGC/CHFP and the Chinese Dietary Reference Intakes (DRIs) specifies daily food and nutrient intake goals for Chinese people. Diet quality scores may be used as a tool to assess and promote adherence to the guidelines and indicate the risk of deviation from normal body weight. The shared principles of different scoring systems suggest that a diet is considered of high quality when composed of fruits and non-starchy vegetables, whole grains, legumes, high-quality proteins, and limiting processed foods and the added sugar, trans fat, empty calories, and sodium associated with them. However, due to the heterogeneity of Chinese populations, there needs to be a careful selection of suitable diet quality scores based on the characteristics of the specific population.

The strength of this review lies in the systematic approach to evaluating the validity and generalizability of different dietary scores in reflecting and predicting weight status focused on the Chinese populations that have distinctive dietary patterns to Western countries and are experiencing nutrition transition during rapid economic development. Many of the cohorts included in the review had large sample sizes. However, there are several limitations. All of the studies conducted regarding dietary scores and weight status were observational, and most of them were cross-sectional. Therefore, the chronological order of events cannot be determined, i.e., it is unclear whether differences in dietary quality predict weight change and weight status over time, or those who are overweight or obese are more likely to focus on dietary improvement as a strategy to lose weight, thereby leading to positive associations between some diet quality indices and weight status [43,44]. It has been demonstrated in some non-Chinese populations that those who lost more weight in weight loss or disease prevention trials had more significant improvements in diet quality scores [76,77]. RCTs are needed to determine whether diet quality improvement as an intervention strategy can facilitate weight control overtime in Chinese populations. The cross-sectional design of current studies may also introduce information bias in dietary data collection, since dietary intake assessment using dietary recalls or FFQ at a single time point may not reflect long-term dietary intake. There is a need for more longitudinal studies with repeated measures of diet quality over time and to discern the chronological order of exposures and outcomes, i.e., whether the diet quality score at baseline could predict weight change and future obesity risk. 

## 5. Conclusions

In conclusion, diet quality scores are associated with weight status and obesity-related outcomes in many studies among Chinese populations. Diet quality scores tailored to the Chinese diet may be more suitable to reflect both undernutrition and obesity risk for Chinese adults in China during a significant nutrition transition. 

## Figures and Tables

**Table 1:** Summary of the characteristics of the included studies.

Reference	Study Design	Number of Participants	Population	Dietary Assessment Method	Index	Outcome	Findings
Stookey et al., 2000 [43]	Cross-sectional	7450	CHNS 1991, aged 20–59 years	3-day 24-h recall	China DQI	BMI, underweight, overweight, obesity	The risk of being underweight decreased and the risk of being overweight increased with each unit increase in DQI total score.
Gao et al., 2008 [36]	Longitudinal	790 (Chinese subgroup, 51.7% females)	Multi-Ethnic Study of Atherosclerosis (MESA), mean age at 62.9 years	FFQ	HEI-05, HEI-90	BMI, WC	Inverse associations between HEI-05 and BMI at baseline or follow-up, no association with WC.
Xu et al., 2015 [53]	Cross-sectional	2745 (1300 males, 1445 females)	Participants aged over 60 in the CHNS 2009	3-day 24-h recall	DBI-07	BMI, underweight	Underweight was associated with lower dietary balance scores.
Neelakantan et al., 2016 [32]	Nested case-control	751 incident cases of AMI (564 nonfatal and 288 fatal) and 1443 matched controls, 35% females in both groups	The Singapore Chinese Health Study, mean age at 59 years	FFQ	AHEI-2010	BMI	BMI did not differ among quartiles of AHEI among controls.
Tian et al., 2016 [50]	Cross-sectional	17,825 (9459 males, 8366 females)	CHNS (2004, 2006, 2009 and 2011), mean age at 45.4 ± 11.9 years	3-day 24-h recall	DDS	BMI, overweight	A positive association between dietary diversity and being overweight in men only.
Huang et al., 2017 [44]	Cross-sectional	13,833 (53% females)	Four waves (2004, 2006, 2009, and 2011) of the CHNS, mean age at 42.7 ± 10.4 years	3-day 24-h recall	China DQI	BMI, overweight, obesity	Higher DQI scores were positively associated with BMI in overweight and obese individuals compared to normal weight.
Wang et al., 2017 [34]	Longitudinal	4734 (2263 males, 2471 females).	Participants who had ≥2 waves of dietary data from 1991 to 2006 in the CHNS, aged 18–65 years	3-day 24-h recall	tAHEI	BMI	No differences in baseline BMI in low versus high tAHEI scores; no differences in BMI in those that had different patterns of tAHEI changes over 1 year.
Yuan et al., 2017 [23]	Cross-sectional	14,584 (52% females)	CHNS 2011	3-day 24-h recall	CHEI	BMI, underweight, overweight, obesity	People who were underweight had lower CHEI scores than people who were normal weight, but no significant difference between normal weight and overweight or obesity.
Zang et al., 2017 [47]	Cross-sectional	1680 (836 males and 844 females)	The Shanghai Diet and Health Survey (SDHS), age ≥ 15 years)	3-day 24-h recall	DBI-07	BMI, obesity	Obese people had higher DQD scores than people with normal weight.
Cheung et al., 2018 [30]	Cross-sectional	211 (115 males and 96 females)	Adults with type 2 diabetes in Hong Kong, China, age 18–65 years	FFQ	AHEI-2010, DASH, DQI-I	BMI, obesity	AHEI-2010, but not DQI-I and DASH, had an inverse association with obesity (BMI ≥ 30 kg/m^2^).
Wang et al., 2018 [35]	Cross-sectional	4440 (2062 males and 2378 females)	CHNS 2006, aged 18–65 years	3-day 24-hr recall	tAHEI; cDGI	BMI, WC	A higher proportion of men had higher BMIs and WCs in the top quintile compared with the bottom quintile of tAHEI scores; no significant differences in BMI across the quintiles of CDGI, yet men in the top quintile had lower odds of abdominal obesity (WC ≥ 90cm) than the bottom quintile.
Whitton et al., 2018 [31]	Cross-sectional	4617 (Chinese subgroup), 56% females	Singapore Multi-Ethnic Cohort study (MEC), mean age at 44 years	FFQ	aHEI-2010, aMED, DASH	BMI, WC	BMI was negatively associated with aHEI and DASH scores, but not aMED.
Zhang et al., 2018 [48]	Cross-sectional	738 (336 males and 402 females)	Participants aged 50–77 years in the 2010–2012 National Nutrition and Health Survey in Yunnan province, southwest China	3-day 24-h recall	DBI-07	BMI, underweight	Underweight people had higher LBS and DQD scores.
Zhao et al., 2018 [51]	Cross-sectional	1520 (527 males and 993 females); 2398 (52.1% females)	Primary dataset: Chinese Urban Adults Diet and Health Study (CUADHS); verification dataset:CHNS 2009	FFQ and 24-h recall	HFD	BMI, WC	Higher HFD was associated with lower WC in the CHNS but not the CUADHS dataset; BMI was not associated with HFD for both datasets.
Chou et al., 2019 [33]	Longitudinal *	436 (195 males and 231 females)	Community-dwelling elders (aged 65 years or older) in Taipei, China	FFQ	mAHEI	BMI	BMI at baseline did not differ between mAHEI tertiles.
Jia et al., 2020 [52]	Cross-sectional	1320 (621 males and 699 females)	Working age (18–60 years) adults in Inner Mongolia, China	3-day 24-h recall	DASH, aMED	BMI, WC, WC/BMI	aMed was inversely associated with WC, BMI, and WC-BMI, while DASH was only associated with WC.
Nguyen et al., 2020 [41]	Longitudinal *	60,161 men and 72,445 women	the Shanghai Women’s Health Study (SWHS) and the Shanghai Men’s Health Study (SMHS)	FFQ	CHFP score	BMI	Baseline BMI did not differ among quartiles of CHFP among participants
Wang et al., 2020 [42]	Longitudinal *	3450 females	5-year breast cancer survivors aged 25–70 years from the Shanghai Breast Cancer Survival Study	FFQ	CHFP scores, modified DASH, and HEI-2015	BMI	Participants within the highest quartile of CHFP-2007 were more likely to have lower BMI than those in the lowest quartile.
Zhou et al., 2020 [49]	Longitudinal *	30,626 (52.8% females)	CHNS (2004, 2006, 2009, and 2011), aged 18–65 years	3-day 24-hr recall	DQD (2)	BMI, Obesity	As BMI increased, the DQD increased; the normal weight group had lower DQD (2) scores than the obesity and overweight groups
Liu et al., 2021 [37]	Longitudinal	6398 (53% females)	CHNS from 1997 to 2015	3-day 24-h recall	CHEI	BMI	Those who had a low score and remained low over time were less likely to have a normal BMI at baseline

* These studies were longitudinal for assessing the relationship between baseline dietary scores and primary outcomes during follow-up, yet the assessment of the relationship between dietary scores and BMI at baseline was cross-sectional. Abbreviations: AHEI: alternative healthy eating index; DASH: dietary approach to stope hypertension; DQI-I: diet quality index-international; DQD: diet quality divergence index; CHEI: Chinese healthy eating index; CDGI: Chinese dietary guidelines index; CHFP: Chinese food pagoda DBI: Diet balance index; DDS: Dietary diversity score; aMED: alternate Mediterranean diet score.

**Table 2:** Characteristics of different dietary indices.

Dietary Index	Reference Guidelines	Food Groups Included	Foods and Nutrients to Limit	Scoring System	Note
**Internationally Used Scores**					
HEI-05 [54]	DGA	Total fruit, whole fruit, total vegetables, dark green and orange vegetables and legumes, total grains, whole grains, milk, meat and beans, oils,	Saturated fat, sodium, and energy from solid fats, alcohol, and added sugars	Sum of score for each food component; scale 0–100	The most updated version of HEI, HEI-2015 has some revisions on the components such as removing alcohol and including a restriction on refined grains.
AHEI [29]	Alternative to the HEI, based on foods and nutrients which can prevent chronic disease risks	Vegetables, fruit, nuts and soy, ratio of white to red meat, total fiber, ratio of polyunsaturated fatty acids (PUFA) to saturated fatty acids (SFA), multivitamin use	Trans fat, alcohol consumption	Sum of score for each food component; scale 0–100	
DASH [55]	the DASH diet	Fruits, vegetables, nuts and seeds and legumes, low-fat dairy, and whole grains	Sodium, sweetened beverages, and red and processed meat	This score is cohort-specific, according to quintile rankings of participants.	
aMed [22]	the Mediterranean diet	Whole grains, vegetables, fruits, legumes, nuts, fish, ratio of monounsaturated fat (MUFA) to SFA	Red and processed meats, alcohol	This score is cohort-specific, according to the median intake of each component in the cohort (0 point for ≤ median and 1 point for > median); total score is the sum of each component score (total scale 0–9)	
		Scores tailored to Chinese			
CHEI [23]	CHFP	Total grains, whole grains and mixed beans, tubers, total vegetables, dark vegetables, fruits, dairy, soybeans, fish and seafood, poultry, eggs, and seeds and nuts	Red meat, cooking oil, sodium, added sugar and alcohol	Sum of score for each food component; scale 0–100	
CHFP [40]	CHFP	Grains, vegetables, fruits, dairy, beans, meat and poultry, fish and shrimp, eggs	Fats and oils, and salt	Sum of score for each food component; scale 0–100	
China DQI [43]	DGC	Diet variety, total carbohydrate, fruit and vegetables, protein calcium, protein	Total fat, SFA, total energy, sodium, and alcoholic beverages	Sum of score for each food component	
DBI [45]	CHFP	Cereals, vegetables and fruits, dairy products, soybean and soybean products, animal food, dietary variety, drinking water	Condiments and alcoholic beverage	With both negative and positive scores indicating inadequacy and excessive intake for each food component, thus the total score converges at 0.	The DBI contains a set of scores: LBS assesses food groups consumption not meeting recommendations; HBS assesses those exceeding recommendations; DQD assesses imbalanced intakes (i.e. the absolute values of LBS + HBS)
DQD [49]	CHFP	Cereal and potatoes, fruits, vegetables, eggs, aquatic products, meat, and poultry, legumes, and nuts, milk, and milk products		This score sums up all the divergences for eight food categories	
DDS [50]		Grains; vegetables; fruits; meat, poultry, and seafood; dairy; and beans, eggs, and nuts		Sum of score for each component; scale 0–6	
HFD [51]		Whole grains and legumes, tubers, other vegetables, vitamin A or C rich vegetables, fruits, dairy, soy products, nuts and seeds, aquatic products, refined grains, meat and poultry, eggs, oils	Salt, added sugar	Scoring is based on an algorithm. The total score ranges from 0 to 1 with 1 being more diverse.	

Abbreviations: AHEI: alternative healthy eating index; DASH: dietary approach to stope hypertension; DQI-I: diet quality index-international; DQD: diet quality divergence index; CHEI: Chinese healthy eating index; CDG: Chinese dietary guidelines; CHFP: Chinese food pagoda; DBI: Diet balance index; DGA: Dietary guidelines for Americans; DDS: Dietary diversity score; aMED: alternate Mediterranean diet score.

## Supplementary Materials

The following are available online at https://www.mdpi.com/article/10.3390/nu13103555/s1, Table S1: The quality score of each study.
