# Supplementary material for: A Systematic Review of Diet Quality Index and Obesity among Chinese Adults
Source: Nutrients. 2021 Oct 11;13(10):3555. doi: 10.3390/nu13103555 (PMC8538294; doi:10.3390/nu13103555)
Supplement: Supplementary file 1 [file nutrients-13-03555-s001.zip › nutrients-1372533-supplementary/Supplementary Table S1.pdf]

Supplementary Table S1. The quality score of each study.

Reference	Quality of Study
Stookey et al., 2000	7
Gao et al., 2008	11
Xu et al., 2015	7
Neelakantan et al., 2016	7
Tian et al., 2016	7
Hunag et al., 2017	7
Wang et al., 2017	9
Yuan et al., 2017	6
Zang et al., 2017	7
Cheung et al., 2018	8
Wang et al., 2018	7
Whitton et al., 2018	7
Zhang et al., 2018	6
Zhao et al., 2018	7
Chou et al., 2019	6
Jia et al., 2020	7
Nguyen et al., 2020	6
Wang et al., 2020	6
Zhou et al., 2020	7
Liu et al., 2021	11
